# Correlation of fragmented QRS with right ventricular indexes and fibrosis in patients with repaired tetralogy of fallot, by cardiac magnetic resonance imaging

**DOI:** 10.1186/1532-429X-17-S1-P215

**Published:** 2015-02-03

**Authors:** Zahra Alizadeh Sani, Ali Vasheghani farahani, Zahra Khajali, Majid Jamshidi, Mahshid Hesami, Hamidreza Fallahabadi, Mousa Alimohammadi, Azin Seifi

**Affiliations:** Rajaie Cardiovascular Medical and Research Center, Iran University of Medical Sciences, Tehran, Iran (the Islamic Republic of; department of medical sciences, Mashhad Branch, Islamic Azad University, Mashhad, Iran (the Islamic Republic of; Electrophysiology Department, Tehran University of Medical Sciences, Tehran, Iran (the Islamic Republic of

## Background

Although surgery have improved the prognosis for patients who repaired tetralogy of Fallot (TOF); late ventricular dysfunction, scar and fibrosis remains a problem which weakens the prognosis. Late gadolinium enhancement (LGE) cardiovascular magnetic resonance (CMR) also shows this scar tissue and fibrosis. In these patients decreasing in the right ventricular (RV) ejection fraction and an increase in end diastolic volume are indications for the pulmonic valve replacement. Fragmented QRS (fQRS) on 12-lead electrocardiogram represents myocardial conduction delays due to myocardial fibrosis.In this study we define the relationship between the fQRS and RV fibrosis, function, volume and the role of fQRS in the follow up and assessment of patients with repaired TOF.

## Methods

32 patients were enrolled in the study and the extent of fQRS in each patient was estimated by counting the number of electrocardiographic leads with QRS. The relation between fQRS and RV indexes and fibrosis in CMR was determined.

## Results

The mean percentage of scar tissue in those who had RVEF< 45% was 24% and 5/8% in patients with RVEF> 45%, that was statistically significant.(P-value= 0.0001). The overall accuracy of the test to identify patients with RVEF<45%, was 98%; that was statistically significant and could be generalized to the target population. (P-value= 0.0001) There was also a meaningful relationship between fQRS and RV end diastolic volume index. (P-value= 0.003) The sensitivity and specificity of fQRS in identifying patients with RVEDVI>150 ml/m^2^ were 87% and 62%, respectively; PPV=70% and NPV=83%.The relation of fQRS and RV end systolic volume index was statistically meaningful. (P-value=0.02)The sensitivity and specificity of fQRS in identifying patients with RVESVI>82 ml/m^2^ were 80% and 66%, respectively; PPV=80% and NPV=66%.The relation of mean right ventricular diastolic and systolic diameter with RVEF was statistically considerable according to the P-value=0.0001 and 0.001, respectively; without any meaningful relationship between RV diastolic diameter and fQRS. (P-value=0.1)And there was relationship between RV diastolic diameter and fQRS. (P-value=0.014)There was a strong inverse linear relationship between the number of fQRS edges and RVEF (r=0.77, P-value=0.0001) in addition to a strong positive linear correlation between the number of edges and the percentage of scar tissue (r=0.88, P-Value=0.001).

## Conclusions

According to this study fQRS could suggest an acceptable screening test in the serial follow up of patients who repaired TOF, considering that CMR was costly and time consuming.

